# Efficacy and Safety of Whey Protein Supplements on Vital Sign and Physical Performance Among Athletes: A Network Meta-Analysis

**DOI:** 10.3389/fphar.2019.00317

**Published:** 2019-04-24

**Authors:** Fui-Ching Lam, Allah Bukhsh, Habib Rehman, Muhammad Khurram Waqas, Nabeel Shahid, Adil Mohammed Khaliel, Ahlam Elhanish, Mustfa Karoud, Ahmed Telb, Tahir Mehmood Khan

**Affiliations:** ^1^School of Pharmacy, Monash University Malaysia, Subang Jaya, Malaysia; ^2^Institute of Pharmaceutical Sciences, University of Veterinary and Animal Sciences, Lahore, Pakistan; ^3^Faculty of Biosciences, University of Veterinary and Animal Sciences, Lahore, Pakistan; ^4^Department of Urology, Bourn Hall Fertility Clinic Dubai, Jumeriah, United Arab Emirates; ^5^Department of Orthopedics, Canadian Specialist Hospital, Abuhail, United Arab Emirates; ^6^Department of Radiology, Emirates Hospital, Jumeriah, United Arab Emirates

**Keywords:** whey protein supplements, network meta-analysis, athletes, vital sign, physical strength, athlete's health and performance

## Abstract

**Introduction:** Athletes train physically to reach beyond their potential maximum aerobic threshold. Whey protein supplements (WPS) are often used in conjunction with physiotherapy and psychotherapy to regain better vital sign and physical performances. This review aimed to explore the clinical evidence on the efficacy and safety of WPS in sports performance and recovery among athletes.

**Methodology:** A comprehensive literature search was performed to identify relevant randomized control trials (RCTs) that investigated the efficacy and safety of WPS on the vital sign and physical performance among athletes. The Cochrane Risk of Bias (ROB) Assessment tools were used to assess the quality of the studies. Meta-analysis was conducted using the frequentist model with STATA version 14.2®.

**Results:** A total of 333,257 research articles were identified out of which 20 RCTs were included for qualitative synthesis and network meta-analysis with 351 participants. Among the studies, 7 had low ROB and 3 RCTs had high ROB. Of these 20 trials, 16 trials were randomized clinical trials which compared whey protein supplements (WPS) with various comparators i.e., L-alanine, bovine colostrum, carbohydrate, casein, leucine, maltodextrin, rice, protein + caffeine were compared with placebo. Analysis from the pairwise meta-analysis revealed that for respiratory exchange ratio (RER) WPS was found to be significantly improving compared to maltodextrin (WMD = 0.012; 95%CI = 0.001, 0.023). Similarity to RPE (Rate Perceived Exertion), slight difference between WPS and the comparators, however, when the estimation was favorable to the comparators, there was moderate-high heterogeneity. For *VO*_2max_, high heterogeneity appeared when WPS compared to maltodextrin with the *I*^2^ = 97.8% (WMD = 4.064; 95% CI = −4.230, 12.359), meanwhile bovine colostrum (WMD = −2.658; 95%CI = −6.180, 0.865) only comparator that was better than WPS. According to the estimated effect of the supplements on physical performance outcome results, maximum power (8 studies, 185 athletes), highest ranked was bovine colostrum (SUCRA = 70.7%) and the lowest ranked was placebo (SUCRA = 17.9%), yet all insignificant. Then again, on average power (nine studies, 187 athletes), WPS was the highest ranked (SUCRA = 75.4 %) about −112.00 watt (−187.91, −36.08) and most of the estimations were significant. Body mass was reported in 10 studies (171 athletes), carbohydrate may be at the highest ranked (SUCRA = 66.9%) but it is insignificant. Thought the second highest ranked was WPS (SUCRA = 64.7%) and it is significant (WMD = −6.89 kg; CI = −8.24, −5.54).

**Conclusion:** The findings of this review support the efficacy and safety of WPS as an ergogenic aid on athletes' sports performance and recovery. The overall quality of clinical evidence was found to be valid and reliable from the comprehensive search strategy and ROB assessment.

## Introduction

Athletes train to be skilful and physically fit to compete and ensure success against their opponents. The effect of athletes' stamina, body structure and skill development are essential to able to do so, while an effective while an effective nutrition and diet plan to ensure good health and well-being of athletes. It is support from the supplement as ergogenic aids to maintain their performance and to gain a competitive edge. The availability and consumption of supplements, along with physiotherapy and psychotherapy, have been recognized as ergogenic advantages in sports performance and recovery (Wiese-Bjornstal, 2010; Chan et al., 2011). supplements might have substances that are harmful and life-threatening effects on athlete health such as alcohol, steroid and caffeine (Silver, 2001).

World Anti-doping Agency (WADA) was established to promote coordinate and monitor illicit drugs use in sports internationally. However, dietary and nutritional supplements have become distressing matters. For many countries and manufacturers of supplements have a lack of quality control, some supplements contain substances that were prohibited such as caffeine and alcohol (Willick et al., 2016). Hence, options for supplements are limited for athletes to compete ethically. One of the popular and easy to purchase protein supplement in sports is whey protein supplements (WPS) as it has shown ergogenic aids which absorbed rapidly, includes all the essential amino acids, and has a high proportion of branched-chain amino acids (MacKenzie-Shalders et al., 2015; Frank et al., 2017).

Various systematic review and meta-analysis published that summaries the effect of whey protein (WP) as a dietary supplement (Nissen and Sharp, 2003; Schoenfeld et al., 2013; Miller et al., 2014). However, there is a lack of consensus over the use of WP, yet, some clinical studies concluded consuming other protein sources or supplements are better than WP (Taylor et al., 2016), which is in contrast with some other studies (Kraemer et al., 2015; Hansen et al., 2016) that support WP in comparison to others. Moreover, the quality of studies and risk of bias is another issue that is often neglected while scrutinizing the evidence of other supplements in comparison to WP. The current systematic review and network meta-analysis aim to explore the clinical efficacy and safety of WPS on athletes' vital sign and physical performance.

## Methods

### Study Design and Selection

A systematic review and network meta-analysis were conducted to identify eligible randomized controlled trials (RCTs). The search strategy used the keyword of “whey^*^” combined individually with “athlete^*^,” “injur^*^,” “muscle^*^,” “perform^*^,” and “recover^*^” on databases as well as specific journals: PubMed, EMBASE via Ovid, Scopus, Cochrane, Cumulative Index to Nursing and Allied Health Literature (CINAHL) via EBSCOhost, SPORTDiscus, Health & Medicine Database via ProQuest, Wiley Online Library, Web of Science, ScienceDirect, Taylor & Francis and SAGE. All experimental and observational studies were considered for potential exclusion in this systematic review. No restriction was placed on language. The searched timeframe was from the date of database inception until December 2017. However, studies design on expert opinions, case reports/series, surveys, review articles, editorials, commercial advertisements, magazine articles, unpublished articles were excluded. The protocol of this study was registered in PROSPERO 2016 and the register identification is CRD42016041842 (Lam et al., 2016) and reported the network meta-analysis according to Preferred Reporting Items for Systematic Reviews and Meta-Analyses (PRISMA) (Moher et al., 2009). Please see Appendix 1 in the Supplementary Material for detailed PRISMA checklist.

### Population of Interest

The participants included were active athletes who experienced fatigue and had recovered and/or had been hindered in their performance. Also, studies that observed on participations who are resistance-trained, trained and physically active were deem be athletes as these participants undertook overpowering physical activities during the intervention that were equivalent to athletes. Regardless of athletes' age and gender. However, studies that observed on retired athletes, mixed athletes with non-athletes, animals, cells, and gels were excluded.

### Interventions

The intervention was WP or supplements containing WP. The intervention was found in the form of isolate, concentrate, hydrolysate, denature, and protein bars.

### Comparators

Comparators were carbohydrate supplements, protein-containing foods from animal sources (e.g., meat, fish, dairy products, and eggs), protein-containing vegetarian sources (e.g., tofu, legumes, and soy protein), vitamins (e.g., multivitamin, vitamin B, beta-carotene, and folic acid), minerals (e.g., calcium, iron and zinc) and placebos (include no treatment and treatment as usual).

### Outcomes Measure

This systematic review and meta-analysis have measured two outcomes which associated with aims: -

- Vital signs of heart rate, respiratory exchange ratio (RER), rate perceived exertion (RPE), and maximum volume of oxygen (*VO*_2max_ );
- Strength and body composition which were maximum power, average power, and body mass.

### Data Extraction

The extracted data were entered into Microsoft Excel 2016, namely (Boutron et al., 2008):

1. General information (first author surname, title, year of publication, journal name).
2. The article study methods and characteristic (study design).
3. Participants (age, gender, weight, heights, and sporting activity).
4. Intervention (dose of WP consumed and number times consumed).
5. Comparators (type, dose, and number times consumed).
6. Outcomes: -
  - Outcomes that contributed to vital sign and physical performances.
  - The data obtained after the participants consumed the intervention or control.
  - Most of the data located within the text of the articles and presented in tabular form or graphs
  - When data was in standard error or standard error mean, it was transformed into a standard deviation (Higgins et al., 2011b).

### Assessment of Risk of Bias for Included Studies

The included studies were assessed for their ROB by two reviewers independently. Both assessment results were compared and verified for accuracy. A Cochrane ROB criteria were used to assess the quality of the RCTs (Higgins et al., 2011a) (Appendix 2.1 in Supplementary Material).

### Statistical Analysis

Two types of meta-analysis were conducted with STATA version 14.2®. Firstly, standard pairwise meta-analysis with a random-effects model (Borenstein et al., 2009) was performed and assessed I-squared (*I*^2^) metrics for heterogeneity. Thus, appearance of heterogeneous when the *I*^2^ appeared to have 50% and above (Higgins et al., 2003). Secondly, random-effect network meta-analysis was performed using frequentist model to compare different interventions with each other. For a common heterogeneity variable for all comparisons was assessed by studies tau-square (**τ**^**2**^) test (Turner et al., 2012). The type of data for the meta-analysis was continuous data, which contained mean, standard deviation and sample size (Saez de Villarreal et al., 2012). The results pooled estimates of weighted mean difference (WMD) at 95% confidence interval (95% CI).

For indirect and mixed comparisons, network meta-analysis was performed to compare different strategies and the meta-analysis assumes transitivity (i.e., learn about supplement A (vs.) supplement B via supplement B) (Salanti, 2012). The transitivity embraced when the direct comparison between supplements do not differ with respect to the distribution of effect modifiers. For example, studies comparing WPS with placebo were similar to studies comparing carbohydrate with placebo in heart rate parameter. The potential effect modifiers for trials in this setting were the duration of intervention, physical activities during intervention duration and dosage of supplements. On the other hand, disagreement between direct and indirect evidence suggests that the transitivity assumption might not be embraced.

Firstly, investigate a loop-specific approach for consistency within every triangular or quadratic loop as the difference between direct and indirect estimates for a specific comparison in the loop (inconsistency factor) (Salanti, 2012) (Veroniki et al., 2013). Secondly, performed the design-by-treatment interaction model and examined chi-square (χ^2^) test for a single inference about the plausibility of assuming consistency throughout the entire network (Higgins et al., 2012).

For a better understanding of intervention, surface under the cumulative ranking curve (SUCRA) probabilities conducted to rank the supplements for an outcome. The larger the SUCRA value (express in percentage) was, the better the rank of the intervention would be (Chaimani et al., 2013).

## Result

### Study Characteristics

**Figure 1:**
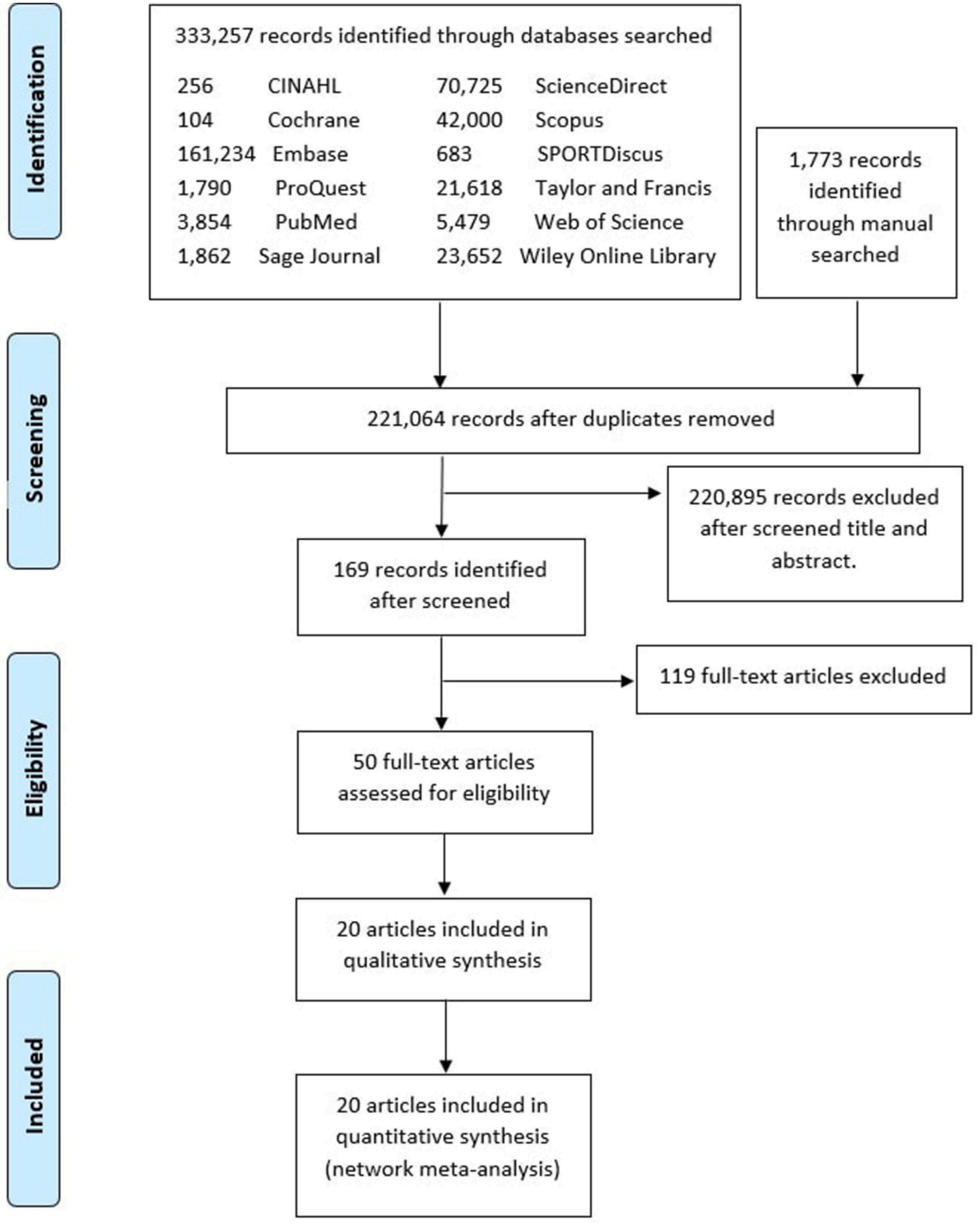
PRISMA flow diagram.

The PRISMA flowchart (Figure 1) shows electronic searching processes. Of 169 potentially relevant articles initially screened, 20 articles with 351 participant athletes met the inclusion criteria. The descriptive study characteristics are presented in (Table 1). All the included articles are 16 RCTs are blinding while four articles are non-blinding (Hoffman et al., 2009; Gunnarsson et al., 2013; Impey et al., 2015; Al-Nawaiseh et al., 2016). The most studies demographic were from United States (6 articles) (Hoffman et al., 2009; Smith et al., 2010; Joy et al., 2013; Schroer et al., 2014; Al-Nawaiseh et al., 2016; Taylor et al., 2016). One study each from China (Li and Zhao, 2007), Finland (Breen et al., 2011), New Zealand (Macdermid and Stannard, 2006), Norway (Vegge et al., 2012), and South Africa (Oosthuyse and Millen, 2016).

**Table 1:** Characteristics of the included studies.

**First Author surname and Year**	**Country of study**	**Study Design**	**Participates**							**Supplement**				**Protocol**		
			**Categories of athletes**	**Average age^1^**	**Average weight (kg)^1^**	**Average height (cm)^1^**	**Male**	**Female**	**Total**	**Intervention group (WP)**	**Number of times to consume WP supplement in a day**	**Control group**	**Number of times to consume supplement in a day**	**Intervention duration (day)**	**Physical activity during intervention duration**	**Consume the supplementation during intervention duration**
Al-Nawaiseh et al., 2016	United States	Random, crossover, counterbalanced	College athletes, college-club athletes	21.5	76.5	NA	11	11	22	23 g with 10.6 g EAA, 7.3 g of conditionally EAA, and 5.6 g of non-EAA ON was mixed with 200 ml of skimmed milk to form a protein shake. 2 oral doses of 1,000 mg of vitamin C (ascorbic acid with citrus bioflavonoids) and 400 IU of vitamin E soft gel capsules (d-a-tocopherol)	3	Placebo (non-treatment)	3	17	Stretch and cycle	Before, during, after physical activity
Breen et al., 2011	Finland	Random, counterbalanced, single-blinding	Cyclists	29	77.2	NA	10	–	10	(1) 10.2 g with 25.4 g carbohydrate dissolved in 250 ml of cold water; (2) 20.4 g with 50.8 g carbohydrate dissolved in 250 ml of cold water	2	(1) 25.2 g of carbohydrate dissolved in 250 ml of cold water, (2) 50.4 g CHO dissolved in 250 ml of cold water	2	28	Cycle	After physical activity
Coombes et al., 2002	Australia	Random, placebo-controlled study, double-blinding	Cyclists	30	74	NA	28	–	28	60 g (pure)	2	60 g bovine colostrum	2	56	Warm up, stretching exercises, cycle	Before and after physical activity
Gunnarsson et al., 2013	Denmark	Random	Soccer players	24	80.5	182	16	–	16	HPC with carbohydrates	1	Placebo (normal diet)	1	2	Soccer game	After physical activity
Hansen et al., 2016	Denmark	Random, block, single-blinding	Elite orienteers	19.5	71.9	183	18	–	18	0.2 g/kg/h with 1 g/kg/h of carbohydrate	4	1.2 g/kg/h of carbohydrate	4	7	Cycle, mix of distance training, interval training, mountain climb	After physical activity
Highton et al., 2013	United Kingdom	Random, crossover, double-blinding	Soccer, rugby union	23.4	75.3	177.5	9	–	9	2% with 6% carbohydrate	5	8% carbohydrate	5	14	Walk and sprint	During physical activity
Hoffman et al., 2009	United States	Random	Resistance-trained (30/33 were college's football)	20.1	99.2	182.1	33	–	33	42 g of a proprietary blend of protein (enzymatically hydrolyzed collagen protein isolate, WPI, and casein protein isolate) with 2 g of carbohydrate	2	Placebo (normal diet)	2	70	High pull, bench press, seated shoulder press, dumbbell shoulder press/behind-the-neck, triceps push-downs, partner neck exercise etc.	Before and after physical activity
Impey et al., 2015	United Kingdom	Random, counterbalanced (Latin Squares approach)	Cyclists and triathletes	29	79.4	179.7	9	–	9	22 g with 2.1 g leucine, 4.9 g BCAA, 9.3 EAA, 500 ml water	1	5 g/kg carbohydrate 2 g/ kg protein, 1 g/kg fat	1	7	Cycle	Before physical activity
Joy et al., 2013	United States	Random, double-blinding	Resistance training experience	21.3	76.1	177.8	24	–	24	48 g	1	48 grams of rice	1	56	Resistance training, cycle test	Before physical activity
Li and Zhao, 2007	China	Random, blinding	Amateur football players	21	64.8	172.4	16	–	16	25 g with 800 ml	2	25 g carbohydrate 800 ml	2	72	Cycle, jump, push up, run	After physical activity
Lollo et al., 2011	Brazil	Random, double-blinding	Soccer players	19	74.4	181.5	24	–	24	91.40%	1	88.6% casein	1	56	Cycle, soccer	After physical activity
Lollo et al., 2014	Brazil	Random, double-blinding	Soccer players	18	74	178.5	24	–	24	0.5 g/kg concentrate	2	Maltodextrin	2	180	Soccer training	Before and after physical activity
Macdermid and Stannard, 2006	New Zealand	Random, balanced order, blinding	Cyclists	33.6	68.6	175.4	7	–	7	1.2–1.4 g/kg/d with carbohydrate intake of 7–10 g/kg	1	Protein intake of 3–4 g/kg/d and a carbohydrate intake of ≤ 5 g/kg	1	16	Cycle	During physical activity
Naclerio et al., 2015	United Kingdom	Random, counterbalanced, cross over, double-blinding	Amateur soccer players	24	77.5	181	16	–	16	14.5 g with multi-ingredient (MTN; carbohydrate (53 g), L-glutamine (5 g), and L-carnitine L-tartrate (1.5 g),	4	69.5 g carbohydrate	4	13	Run, jog, run	Before, during, after physical activity
Oosthuyse and Millen, 2016	South Africa	Random, four way crossover, double-blinding	Cyclists	38.9	78.5	179.8	8	-	8	Carbohydrate-whey hydrolysate	3	Carbohydrate	3	30	Cycle	Before and during physical activity
Schroer et al., 2014	United States	Random, counterbalanced, placebo-controlled, double-blinding	Cyclists	22.3	70	167	4	4	8	45 g/L	Every 15 min	15 g/ of L-alanine	Every 15 minutes	16	Cycle	Before and after physical activity
Shing et al., 2006	Australia	Random, placebo controlled, double-blinding	Road cyclists	28	76.4	179.9	29	-	29	10 g with 50 ml water and 100 ml skim milk	1	10 g Intact bovine CPC, 50 ml water and 100 ml skim milk	1	70	Cycle	Unknown
Smith et al., 2010	United States	Random, placebo controlled parallel, single-blinding	Moderately-trained	21.1	66.2	173.4	24		24	8 g with cholesterol, sodium carbohydrates, sugar, vitamin A, C B12, B6	1	Maltodextrin: 17 g	1	21	Run	Before physical activity
Taylor et al., 2016	United States	Random, double-blinding	Basketball players	20.5	67.1	169.5		14	14	24 g with in water	2	24 g of maltodextrin	2	56	Lower body resistance, “explosive” exercises such as squat jumps, push jerks, and hang cleans), training drills	Before and after physical activity
Vegge et al., 2012	Norway	Random, crossover, double-blinding	Cyclists	22	NA	NA	12	–	12	15.3 g/h with maltodextrin	1	60 g/h maltodextrin	1	60	Cycle	During physical activity

1 *Mean ± Standard Deviation*.

*NA, not available*.

Additionally, the researchers who were consistent in publishing the most regarding WPS for athletes were Lollo and colleagues, had two publications (Lollo et al., 2011, 2014). Two articles have both males and female athletes (Schroer et al., 2014; Al-Nawaiseh et al., 2016), an article has only female athletes (Taylor et al., 2016) while an article was not taken account regarding their gender (Fukuda et al., 2010). In total 351 athletes participated: 298 males, 29 females and 24 athletes did not categories. Furthermore, the minimum number of participants in a study among the included for this review was *n* = 7 (Macdermid and Stannard, 2006) and the maximum was *n* = 33 participants (Hoffman et al., 2009).

WPS and the comparators (L-alanine, bovine colostrum, carbohydrate, casein, leucine, maltodextrin, rice, protein + caffeine) were compared with placebo. The number of participants based on the analyses indicated that a total of 351 athletes consumed WPS, followed by carbohydrate (154 athletes), and placebo (137 athletes), respectively. While protein + caffeine (9 athletes) as well as leucine (9 athletes) had the smallest number of participants. The results of network meta-analysis showed in terms of efficacy in Table 2 on the respective outcomes.

**Table 2:** Results of network meta-analyses.

**a) HEART RATE (bpm)**						
**WPS**						
0.88 (−14.82, 16.58)	**L-alanine**					
4.94 (−5.19, 15.07)	3.06 (−7.07, 13.19)	**Carbohydrate**				
1.88 (−15.48, 19.24)	−2.79 (−20.20, 14.62)	−3.06 (−18.86, 12.74)	**Casein**			
0.88 (−16.24, 18.00)	−0.00 (−12.12, 12.12)	−4.06 (−19.60, 11.48)	−1.00 (−9.34, 7.34)	**Leucine**		
−1.00 (−2.09, 0.09)	−1.00 (−12.78, 10.78)	−5.94 (−16.13, 4.25)	−2.88 (−20.27, 14.51)	−1.88 (−19.03, 15.28)	**Maltodextrin**	
1.00 (−8.60, 10.60)	−2.88 (−15.35, 9.59)	−3.94 (−17.90, 10.02)	−0.88 (−20.72, 18.96)	0.12 (−19.51, 19.75)	2.00 (−7.66, 11.66)	**Placebo**
**b) RESPIRATORY EXCHANGE RATION**						
**WPS**						
0.01 (−0.01, 0.03)	**L-alanine**					
−0.00 (−0.03, 0.03)	−0.01 (−0.03, 0.01)	**Carbohydrate**				
0.00 (−0.00, 0.00)	−0.01 (−0.04, 0.02)	0.00 (−0.03, 0.03)	**Maltodextrin**			
0.02 (−0.01, 0.05)	0.01 (−0.03, 0.05)	0.02 (−0.02, 0.06)	0.02 (−0.01, 0.05)	**Placebo**		
**c) RATE PERCEIVED EXERTION**						
**WPS**						
−2.00 (−3.54, −0.46)	L–alanine					
0.00 (−1.97, 1.97)	0.10 (−1.84, 2.04)	**Carbohydrate**				
0.60 (−2.10, 3.31)	−0.12 (−3.07, 2.83)	0.60 (−2.11, 3.31)	**Placebo**			
1.90 (−0.56, 4.37)	0.70 (−1.20, 2.60)	1.90 (−0.57, 4.37)	1.30 (−1.14, 3.74)	**Protein + Caffeine**		
**d) MAXIMUM VOLUME OF OXYGEN (ml/kg/min)**						
**WPS**						
1.56 (−30.75, 33.87)	**L-alanine**					
44.84 (−210.49, 300.16)	43.27 (−211.92, 298.46)	**Bovine colostrum**				
−3.99 (−18.53, 10.55)	−5.55 (−40.95, 29.84)	−48.83 (−304.56, 206.91)	**Carbohydrate**			
0.07 (−13.63, 13.77)	−1.50 (−35.30, 32.30)	−44.77 (−300.37, 210.83)	4.06 (−15.92, 24.03)	**Maltodextrin**		
5.72 (−4.08, 15.52)	4.16 (−28.88, 37.19)	−39.12 (−294.58, 216.35)	9.71 (−7.82, 27.24)	5.65 (−11.13, 22.44)	**Placebo**	
**e) MAXIMUM POWER (WATT)**						
**WPS**						
66.64 (−135.37, 268.65)	**Bovine colostrum**					
14.38 (−249.13, 277.89)	−52.25 (−383.51, 279.00)	**Carbohydrate**				
−91.78 (−250.97, 67.41)	−158.41 (−414.78, 97.95)	−106.16 (−413.99, 201.67)	**Placebo**			
31.14 (−245.32, 307.60)	−35.49 (−377.79, 306.81)	16.76 (−365.17, 398.69)	122.92 (−196.09, 441.93)	**Rice**		
**f) AVERAGE POWER (WATT)**						
**WPS**						
0.44 (−22.32, 23.20)	**Bovine colostrum**					
−112.00 (−187.91, −36.08)	−112.43 (−191.69, −33.18)	**Carbohydrate**				
−2.03 (−7.00, 2.93)	−2.47 (−25.34, 20.39)	109.96 (33.88, 186.04)	**Placebo**			
**g) BODY MASS (kg)**						
**WPS**						
1.50 (−20.39, 23.40)	**Bovine colostrum**					
2.00 (−10.40, 14.40)	0.50 (−24.66, 25.66)	**Carbohydrate**				
0.20 (−26.06, 26.46)	−1.30 (−35.48, 32.88)	−1.80 (−30.84, 27.24)	**Casein**			
−6.89 (−8.24, −5.54)	−8.39 (−30.32, 13.54)	−8.89 (−21.36, 3.58)	−7.09 (−33.38, 19.20)	**Maltodextrin**		
−1.96 (−4.76, 0.84)	−3.46 (−25.53, 18.61)	−3.96 (−16.67, 8.75)	−2.16 (−28.57, 24.25)	4.93 (1.82, 8.04)	**Placebo**	

*Estimates are presented as WMD and 95% confidence intervals. Comparisons between supplements should be read from column to row. The estimate is in a cell between the column-defining supplement and the row-defining supplement. When WMD lower than 0, it is favor to the column-defining supplement. Statistically significant values have been bold and underlined*.

The shortest intervention duration, on average, was 2 days (Gunnarsson et al., 2013) and the longest duration was 180 days (Lollo et al., 2014). Participants consumed supplements, in extreme cases; one study has participants taking supplements every 15 min (Schroer et al., 2014). Participants consumed supplements before, during and/or after physical activities.

### Risk of Bias

**Figure 2:**
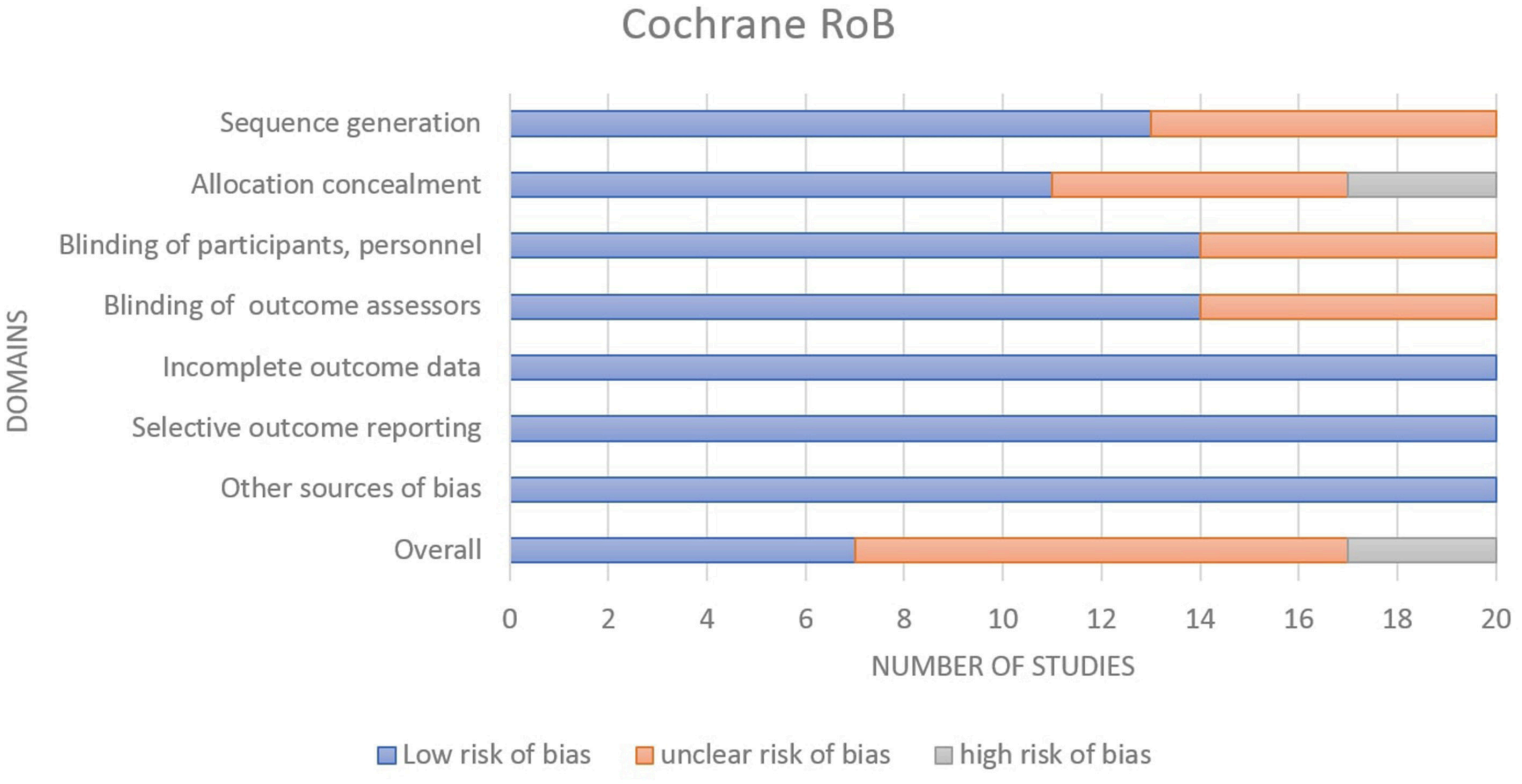
Summary of Cochrane risk of Bias for the RCTs.

A total of 20 RCTs were assessed using the Cochrane ROB Tools assessment (Appendix 2.2 in Supplementary Material). The summary of Cochrane ROB for RCTs (Figure 2) shows 7 studies (35%) have overall low ROB, 10 studies (50%) have overall unclear ROB and 3 studies (15%) have overall high ROB (Smith et al., 2010; Breen et al., 2011;Hansen et al., 2016).

**Figure 3:**
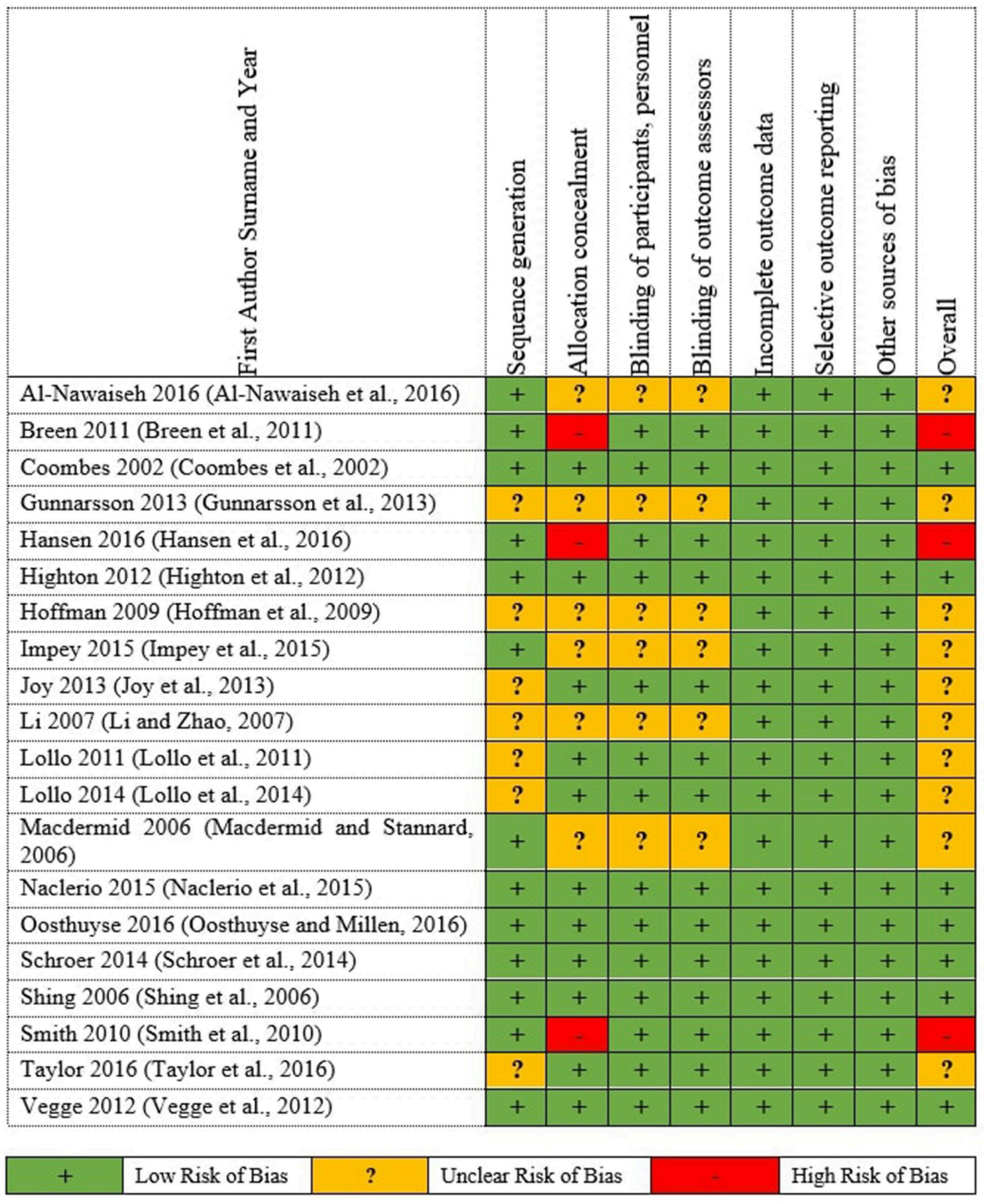
Summary of Cochrane risk of Bias for the individual RCTs.

Moreover, the high ROB distributed only at that allocation concealment shows on Figure 3 of the individual studies ROB. The cause of the high ROB was single blinding conducted in the 4 studies, thus, either participants or investigators could possibly foresee assignments and impact on participants' behavior and participation and outcome assessment. While, 20 studies (100%) low ROB for incomplete outcome data, selective outcome reporting and other sources of bias domains.

### Meta-Analysis

The pairwise comparisons of the efficacy of WPS as compared to other supplements on vital sign and physical performances among athletes (Appendix 3 in Supplementary Material). In terms of the efficacy on vital sign outcome, the analysis result shows heart rate (bpm) slightly increases and decreases. The highest heart rate is 5 bpm (95%CI = −5.231, 15.231) favorable to L-alanine, and lowest heart rate is −1 bpm (95%CI = −2.089, 0.089) favorable to protein + caffeine, yet not significant. For RER, all slightly favorable to WPS compared to the comparators, and it is significant when WPS compared to maltodextrin (WMD = 0.012; 95%CI = 0.001, 0.023). Similarity to RPE, slight difference between WPS and the comparators, however, when the estimation was favorable to the comparators, there was moderate-high heterogeneity. For *VO*_2max_, high heterogeneity appeared when WPS compared to maltodextrin with the *I*^2^ = 97.8% (WMD = 4.064; 95%CI = −4.230, 12.359), meanwhile bovine colostrum (WMD = −2.658; 95%CI = −6.180, 0.865) only comparator that was better than WPS. Apart from RPE and *VO*_2max_ on WPS against maltodextrin have heterogeneity, no evidence of heterogeneity was seen in general.

In terms of the efficacy on physical performances outcome, maximum, and average power (watt) results favorable to WPS and show no heterogeneity but favorable to the comparators show moderate-high heterogeneity, yet not significant. Body mass (kg) parameter has slightly different but no significant between WPS and the comparators; the highest body mass was 0.585 kg (95%CI = −6.122, 7.292) compare to bovine colostrum while the lowest body mass was casein of −5.593 kg (95%CI = −8.131, −3.054; *I*^2^ = 86.0%) with high heterogeneity. Detailed on the results of pairwise meta-analyses are given in the Appendix 3 (Supplementary Material).

In the results of network meta-analysis, statistical heterogeneity found moderate only in the network of RPE on a triangular loop of evidence including supplementation comparison between carbohydrate, combined placebo and WPS (carbohydrate—placebo—WPS) (For Network meta-analysis plots please see Appendix 4 in Supplementary Material). Besides that, no appearance of statistical heterogeneity was seen throughout (Appendixes 5 and 6 in Supplementary Material). The estimates from inconsistency factor (IF) did not show evidence of statistical inconsistency. Moreover, the physical performance outcome shows no triangular or quadratic loops found.

The direct comparisons and network estimates for both vital sign and physical performances outcomes are shown in the league table (Table 2). The comparative efficacy of all supplements ranked with SUCRA probabilities (Appendix 7 in Supplementary Material). For the vital sign outcome result, heart rate was reported in 12 studies (216 athletes), the highest SUCRA ranked was carbohydrate of 74.9% (WMD = −5.94 bpm; 95%CI = −16.13, 4.25) and the lowest ranked was WPS of 33.1% (WMD = 4.94 bpm; 95%CI = −5.19, 15.07) were not significant. RER was reported in five studies (100 athletes), although L-alanine had the highest (SUCRA = 71.1%) ranked while placebo had the lowest (SUCRA = 42.8%) ranked, the estimations of supplements were generally similar to placebo and non-significant (Table 2b). RPE (eight studies, 170 athletes) was superior to protein + caffeine (SUCRA = 93%) yet insignificant, whereas WPS may be at the low ranked (32.7%) but it was significantly lower (WMD = −2.00; 95%CI −3.54, −0.46) of RPE level compared to L-alanine (Table 2c). For *VO*_2max_ (nine studies, 190 athletes), the highest ranked was placebo (SUCRA = 69.4%) and the lowest ranked was carbohydrate (SUCRA = 29.8%). However, it was revealed from the results of NMA that bovine colostrum had the highest rate of oxygen consumption attainable during the incremental or intensity of physical activities, yet not significant (Table 2d).

According to the estimated effect of the supplements on physical performance outcome results (showed in Tables 2e-g), maximum power (8 studies, 185 athletes), highest ranked was bovine colostrum (SUCRA = 70.7%) and the lowest ranked was placebo (SUCRA = 17.9%), yet all insignificant. Then again, on average power (nine studies, 187 athletes), WPS was the highest ranked (SUCRA = 75.4 %) about −112.00 watt (−187.91, −36.08) and most of the estimations were significant. While, the lowest ranked was carbohydrate (SUCRA = 0.2%). Body mass was reported in 10 studies (171 athletes), carbohydrate may be at the highest ranked (SUCRA = 66.9%) but it is insignificant. Thought the second highest ranked was WPS (SUCRA = 64.7%) and it is significant (WMD = −6.89 kg; CI = −8.24, −5.54). While, the lowest ranked was maltodextrin (12.4%).

## Discussion

### Quality of the Studies

The search strategy was robust and unlikely to have missed eligible studies. Majority of the studies had low and unclear ROB. This could due to the methodological difference (may know as methodological heterogeneity), such as binding allocation, a washout period of time and data analysis strategy. For instance, Hoffman et al. (2009) had 70 days of intervention period that was without blinding and had washout period while Breen et al. (2011) on 28 days of intervention period that was single-blinding and no washout period.

### Vital Signs Outcome

#### Heart Rate

Heart rate for athletes is an instrument to determine and monitor their daily effort for every training and how hard their body is being trained. A slower increase in heart rate while training sessions act as proof that athletes are physically fit (Aubert et al., 2003; Li and Kim, 2017). Although a slower heart rate is preferable, the small differences between the comparators have indicated that WPS is capable and comparable to the comparators.

Rapid absorption of fluids and nutrition assist in better cardiovascular performance in athletes (Oosthuyse and Millen, 2016). These twelve studies have individually shown that WPS and comparators were comparably absorbed rapidly. For WPS, it is known to be absorbed more rapidly than most of the other protein sources, thus it appears to resist coagulation in the stomach and surpass intestines relatively fast (Frank et al., 2017). Whereby, Breen et al. (2011), Li and Zhao (2007) and Impey et al. (2015) studies have individually examined slower heart rate in WPS as compared to carbohydrate supplements.

Moreover, Oosthuyse and Millen (2016) studied specifically the effect of supplements (WPS and comparators) and placebo. This study had the carbohydrate-casein only supplement that intended to maintain all measures of systolic function, yet, these supplements during the intervention period were parallel consistently ingestion. Thus, the benefits associated with consumption of WPS in the context of heart rate may not be significant in contrast to athletes who consumed comparators (carbohydrate, casein, L-alanine, maltodextrin supplements and placebo).

Based on these findings on examined all supplements had similar heart rate results, therefore, WPS is capable to act as ergogenic aids in athletes' heart rate. Nevertheless, athletes must be mindful about continuous of having low heart rates as their heart enlarged over a prolonged period of time (Dixon et al., 1992; Imai et al., 1994). This may lead to suffering from athletic heart syndrome and they may need pacemaker later in their lives.

#### Respiratory Exchange Ratio (RER)

Respiratory exchange ratio is one of the most metabolic measurements that indicates fuel (mainly carbohydrate or lipid) is being metabolized to supply the body with energy. When RER value is high, carbohydrates are being utilized, whereas when RER value is low, lipid oxidation is being enhanced (Bergman and Brooks, 1999). Furthermore, the individual studies of RER source data are between 0.8 and 0.9 which corresponds to 50% fat and 50% carbohydrate metabolism (Nelson et al., 2015). Vegge et al. (2012) reported that WPS and maltodextrin supplements were associated RER and had similar RER values throughout the prolonged submaximal exercise, while Schroer et al. (2014) found that WPS did not influence RER or performance. Surprisingly, Breen et al. (2011) found that RER value was not extraordinary high with carbohydrate containing supplements, though the study has high ROB (Figure 3). Therefore, athletes consumed WPS has contributed to a higher RER value for better generation of energy.

#### Rate Perceived Exertion (RPE)

Rate perceived exertion is a method to quantify internal training load or intensity of exercise for athletes. Normally, it is a scale measurement that runs from 0 to 10 rating. Whereby, 0 is no training done and 10 extremely heavy training that athletes are able to cope (Ekblom and Golobarg, 1971; Amtmann et al., 2008; Iellamo et al., 2014). There is a slight difference between the supplements and a study has high ROB (Breen et al., 2011) (Figure 3). Moreover, Highton et al. (2013) reported that athletes who consumed WPS were exercising at a higher exercise intensity compare to carbohydrate, yet for both groups, RPE value had no great difference. Additionally, Naclerio et al. (2015) examined that WPS provided lower RPE values at the beginning and toward the end of soccer compared to carbohydrates alone or a low calorie placebo. The low RPE, especially at the end of exercising sessions, suggested that availability of glycogen would attenuate the rise in fatigue (Naclerio et al., 2015). This indicates that WPS group had lower RPE compared to comparators with the similar workload done. Hence, athletes who consumed WPS were able to have lower RPE and better coping with the intensity of physical exercise.

#### Maximum Volume of Oxygen (***VO***_**2max**_)

Maximum volume of oxygen is defined as the highest rate of oxygen consumption attainable during the incremental or intensity of physical activities (Dlugosz et al., 2013). It also reflects the cardiorespiratory fitness associated with endurance capacity during the prolonged physical activities (Ross et al., 2016). In general, if athletes are performing more intensely, higher will the *VO*_2max_ consumption (Dlugosz et al., 2013). Although the network meta-analysis showed bovine colostrum had better efficacy among all the supplements, Coombes et al. (2002) studied that WPS had similar performance benefits with bovine colostrum alone. Similar to Shing et al. (2006) and Schroer et al. (2014) examined that at the beginning of intensity, there may variance in the intake *VO*_2max_, but, at longer duration, there was no difference in improving intake of oxygen and performance. Thus, this might be one of causes to the huge amount of 95% CI in the network meta-analysis. On the other hand, Smith et al. (2010) studied that 90–115% of *VO*_2max_ for higher-intensity exercise, while consuming caffeine supplementation. Although, the study may have increase the performance, caffeine is an illegal substance that prohibited by WADA (World Anti-Doping Agency, 2017). With these findings, WPS has better ergogenic effect in *VO*_2max_ that allows athletes to have cardiorespiratory fitness while performing intensively.

### Physical Performance

#### Maximum and Average Power

To perform in sport, strength is key performance measurement and one of the main interest that athletes seek for ergogenic aids (Lemon et al., 1992; Tarnopolsky et al., 1992; Al-Nawaiseh et al., 2016). The ergogenic effect in maximum and average power were higher in placebo and carbohydrates, respectively, as compare bovine colostrum. According to the included studies, WPS is comparable as ergogenic aids in strength for athletes as there are only slight differences between WPS and the comparators (Coombes et al., 2002; Hoffman et al., 2009; Joy et al., 2013; Hansen et al., 2016). Moreover, Shing et al. (2006) examined that athletes who consumed WPS experienced a decrease in strength in the beginning, but they recovered from any residual fatigue and remained unchanged at following the 5–6 days. Moreover, Highton et al. (2013) discovered that WPS ingestion enabled a small increase in exercise intensity in the latter stages of the sports exercise compared to carbohydrate. Al-Nawaiseh et al. (2016) also investigated that average power recovered better and managed about 4 times higher for athletes who consumed WPS than placebo. Hence, WPS would assist athletes in strength at a longer period of consumption with the physical activities.

#### Body Mass

The key objectives of athletes' development and well-being are body composition. One of the important body composition measurement is body mass (Anding and Oliver, 2015). The analysis had illustrated that WPS improved athletes' body mass by lowering their body mass better than the competitors, though with a marginal difference. Additionally, the individual studies explained that WPS is an ergogenic aid to the body composition as a whole. The relationship of WPS with body mass is well studied and elaborated by Lollo et al. (2011), Lollo et al. (2014). Additionally, Lollo et al. (2011) have examined that WPS provided an additional benefit for maintaining and gaining muscle mass in athletes, while Lollo et al. (2014) further assessed that WPS has a net effect on muscle mass gain over prolonged exercise. Moreover, Taylor et al. (2016) reported in particular for female athletes who improved lean body mass and reduction in fat mass. Thus, the results suggested that athletes who need to have achieved Ideal weight by losing their body mass for the sports performance are encouraged to consumed WPS while maintaining or gaining muscle mass (Brukner and Khan, 2009).

### Safety

There was no relevant data available on the safety, and no side effect was reported in all of the included studies. Therefore, this systematic review and network meta-analysis study are not in the position to discuss it. Nonetheless, WP is recognized as safe supplements for athletes (Tipton et al., 2004; Bolster et al., 2005), concern arises from WADA insight whereby illegal substances can be found in the interventions from the included studies which are Smith et al. (2010) contained caffeine. Hence, athletes shall be cautious while taking supplements in the content of not violating WADA rule and regulation (World Anti-Doping Agency, 2017).

### Limitation

Several limitations of this systematic review and meta-analysis are worth considering. Foremost, the study did not overcome problems that were inherent in the primary studies. Also, the review did not correct the biases of the primary studies (Garg et al., 2008). Besides, there would have imprecision related to the impossibility of generalizing diverse characteristics from study to study such as age, gender or geographic factors (Higgins and Green, 2011). On top of that, the discussion and conclusion draw from this systematic review and meta-analysis upon the sports performance and recovery among athletes are at the time they were measured. Therefore, this review cannot establish the causation between the parameters and long-term performances and recovery progress for athletes.

### Recommendations

Future directions for research and conducting research that includes larger sample sizes, the inclusion of both gender (especially on female athletes), ages, geographical, type of sport and categories of athletes. Interventions that are consumed before, during and/or after sports performances and recovery process also deserve further considering the effectiveness of improving athletes' vital signs and physical performances. Additionally, follow-up studies could establish effectiveness for the relation between interventions and long-term on vital signs and physical performances progress for athletes. Importantly, it is highly recommended for athletes and their providers are well-inform and updated on WADA guidelines that updated annually before consuming any WPS. These findings are worthy of further inquiry and investigation.

## Conclusion

The systematic review and network meta-analysis study has attempted on the clinical evidence efficacy and safety of WPS on performance and recovery among athletes are promising. First of all, the quality of studies has delivered assure validity and reliability of the clinical evidence. Whereby, all the studies were RCTs, thus, many sources of biases have omitted. Therefore, athletes and multidisciplinary team that manages athlete's health and performance can have a clear directive on the evidence with regards WPS as compared to other protein supplements for a vital sign and physical performance. Besides, the included studies examined as close as possible to real life conditions of sports performances and competition for athletes. Therefore, the study can be used as a guide for better decision-making especially when working with a multidisciplinary approach.

## Author Contributions

TK performed Statistical analysis and interpretation of results. F-CL wrote the initial draft. AB finalized the paper. HR, MKW, NS, AMK, AE, MK, and AT helped in making revisions and finalization of the manuscript.

### Conflict of Interest Statement

The authors declare that the research was conducted in the absence of any commercial or financial relationships that could be construed as a potential conflict of interest.
