# Supplementary material for: Efficacy and Safety of Whey Protein Supplements on Vital Sign and Physical Performance Among Athletes: A Network Meta-Analysis
Source: Front Pharmacol. 2019 Apr 24;10:317. doi: 10.3389/fphar.2019.00317 (PMC6491698; doi:10.3389/fphar.2019.00317)
Supplement: Supplementary file 1 [file Data_Sheet_1.docx]

**Title: Efficacy and safety of whey protein supplements on vital sign and physical performance among athletes: A network meta-analysis**

**SUPPLEMENTARY APPENDIX**

**Contents Page N0**

**Appendix 1:** PRISMA Checklist 2

**Appendix 2:** Cochrane risk of bias tool for RCTs 4

**Appendix 2.1**: Guideline 4

**Appendix 2.2**: The assessment judgment outcomes of RCTs 6

**Appendix 3**: Results of pairwise meta-analysis 16

**Appendix 4**: Network meta-analysis plots 18

**Appendix 5**: Assessment of inconsistency for each outcome network meta-analysis 20

**Appendix 6**: Assessment of global inconsistency in networks using the ‘design-by-treatment’ interaction model 21

**Appendix 7:** SUCRA ranks 22

# Appendix 1: PRISMA Checklist

| **Section/topic** | **#** | **Checklist item** | **Reported on page #** |
| --- | --- | --- | --- |
| **TITLE** | | |  |
| Title | 1 | Identify the report as a systematic review, meta-analysis, or both. | 1 |
| **ABSTRACT** | | |  |
| Structured summary | 2 | Provide a structured summary including, as applicable: background; objectives; data sources; study eligibility criteria, participants, and interventions; study appraisal and synthesis methods; results; limitations; conclusions and implications of key findings; systematic review registration number. | 2 |
| **INTRODUCTION** | | |  |
| Rationale | 3 | Describe the rationale for the review in the context of what is already known. | 3 |
| Objectives | 4 | Provide an explicit statement of questions being addressed with reference to participants, interventions, comparisons, outcomes, and study design (PICOS). | 3 |
| **METHODS** | | |  |
| Protocol and registration | 5 | Indicate if a review protocol exists, if and where it can be accessed (e.g., Web address), and, if available, provide registration information including registration number. | 3-5 |
| Eligibility criteria | 6 | Specify study characteristics (e.g., PICOS, length of follow-up) and report characteristics (e.g., years considered, language, publication status) used as criteria for eligibility, giving rationale. | 3-5 |
| Information sources | 7 | Describe all information sources (e.g., databases with dates of coverage, contact with study authors to identify additional studies) in the search and date last searched. | 3-5 |
| Search | 8 | Present full electronic search strategy for at least one database, including any limits used, such that it could be repeated. | 3-5 |
| Study selection | 9 | State the process for selecting studies (i.e., screening, eligibility, included in systematic review, and, if applicable, included in the meta-analysis). | 3-5 |
| Data collection process | 10 | Describe method of data extraction from reports (e.g., piloted forms, independently, in duplicate) and any processes for obtaining and confirming data from investigators. | 3-5 |
| Data items | 11 | List and define all variables for which data were sought (e.g., PICOS, funding sources) and any assumptions and simplifications made. | 3-5 |
| Risk of bias in individual studies | 12 | Describe methods used for assessing risk of bias of individual studies (including specification of whether this was done at the study or outcome level), and how this information is to be used in any data synthesis. | 3-5 |
| Summary measures | 13 | State the principal summary measures (e.g., risk ratio, difference in means). | 3-5 |

| **Section/topic** | **#** | **Checklist item** | **Reported on page #** |
| --- | --- | --- | --- |
| Synthesis of results | 14 | Describe the methods of handling data and combining results of studies, if done, including measures of consistency (e.g., I^2^) for each meta-analysis. | 3-5 |
| Risk of bias across studies | 15 | Specify any assessment of risk of bias that may affect the cumulative evidence (e.g., publication bias, selective reporting within studies). | 3-5 |
| Additional analyses | 16 | Describe methods of additional analyses (e.g., sensitivity or subgroup analyses, meta-regression), if done, indicating which were pre-specified. | Not Applicable |
| **RESULTS** | | |  |
| Study selection | 17 | Give numbers of studies screened, assessed for eligibility, and included in the review, with reasons for exclusions at each stage, ideally with a flow diagram. | 5-14 |
| Study characteristics | 18 | For each study, present characteristics for which data were extracted (e.g., study size, PICOS, follow-up period) and provide the citations. | 5-14 |
| Risk of bias within studies | 19 | Present data on risk of bias of each study and, if available, any outcome level assessment (see item 12). | 5-14 |
| Results of individual studies | 20 | For all outcomes considered (benefits or harms), present, for each study: (a) simple summary data for each intervention group (b) effect estimates and confidence intervals, ideally with a forest plot. | 5-14 |
| Synthesis of results | 21 | Present results of each meta-analysis done, including confidence intervals and measures of consistency. | 5-14 |
| Risk of bias across studies | 22 | Present results of any assessment of risk of bias across studies (see Item 15). | 5-14 |
| Additional analysis | 23 | Give results of additional analyses, if done (e.g., sensitivity or subgroup analyses, meta-regression [see Item 16]). | Not Applicable |
| **DISCUSSION** | | |  |
| Summary of evidence | 24 | Summarize the main findings including the strength of evidence for each main outcome; consider their relevance to key groups (e.g., healthcare providers, users, and policy makers). | 14-16 |
| Limitations | 25 | Discuss limitations at study and outcome level (e.g., risk of bias), and at review-level (e.g., incomplete retrieval of identified research, reporting bias). | 16 |
| Conclusions | 26 | Provide a general interpretation of the results in the context of other evidence, and implications for future research. | 17 |
| **FUNDING** | | |  |
| Funding | 27 | Describe sources of funding for the systematic review and other support (e.g., supply of data); role of funders for the systematic review. | Not Applicable |

Source from Moher and colleague (2009) [1].

# Appendix 2: Cochrane risk of bias tool for RCTs

## Appendix 2.1: Guideline

Use the modified Cochrane Collaboration tool to assess risk of bias for randomized controlled trials. Bias is assessed as a judgment (high, low, or unclear) for individual elements from five domains (selection, performance, attrition, reporting, and other) [2].

| **Domain** | **Description** | **High Risk of Bias** | **Low Risk of Bias** | **Unclear Risk of Bias** | **Reviewer Assessment** |
| --- | --- | --- | --- | --- | --- |
| Selection bias  **Random sequence generation** | Described the method used to generate the allocation sequence in  sufficient detail to  allow an assessment of whether it should  produce comparable groups | Selection bias (biased allocation to interventions) due to inadequate generation of a randomized sequence | Random sequence generation method should produce comparable groups | Not described in sufficient detail | High  Low  Unclear |
| Selection bias **Allocation concealment** | Described the method used to conceal the allocation  sequence in  sufficient detail to determine whether intervention allocations could have been foreseen before or during enrollment | Selection bias (biased allocation to interventions) due to inadequate concealment of allocations prior to assignment | Intervention allocations  likely could not have been foreseen in before or during enrollment | Not described in sufficient detail | High  Low  Unclear |
| Reporting bias **Selective reporting** | Stated how the possibility of selective outcome reporting was examined by the authors and what was found | Reporting bias  due to selective outcome reporting | Selective outcome  reporting bias not detected | Insufficient information to permit judgment† | High  Low  Unclear |
| Other bias  **Other sources of bias** | Any important concerns about bias not addressed above* | Bias due to problems not covered elsewhere in the table | No other bias detected | There may be a risk of bias, but there is either insufficient information to assess whether an important risk of bias exists or insufficient rationale or evidence that an  identified problem will introduce bias | High  Low  Unclear |

| Performance bias  **Blinding (participants**  **and personnel)** | Described all measures used, if any, to blind study participants and personnel from knowledge of which intervention a participant received. Provided any information relating to whether the intended blinding was effective. | Performance bias due to knowledge of the allocated interventions by participants and personnel during the study. | Blinding was likely effective. | Not described in sufficient detail | High  Low  Unclear |  |
| --- | --- | --- | --- | --- | --- | --- |
| Detection bias  **Blinding (outcome assessment)** | Described all measures used, if  any, to blind outcome assessors from knowledge of which intervention a participant received. Provided any information relating to whether the intended blinding was effective. | Detection bias due to knowledge of the allocated  interventions by outcome assessors. | Blinding was likely effective. | Not described in sufficient detail | High  Low  Unclear |  |
| Attrition bias  **Incomplete outcome data** | Described the completeness of outcome data for each main outcome, including  attrition and exclusions from the analysis. Stated whether attrition and exclusions were reported, the numbers in each intervention group (compared with total randomized participants), reasons for attrition/exclusions where reported. | Attrition bias due to amount, nature or handling of incomplete outcome data. | Handling of incomplete outcome data was complete and unlikely to have produced bias | Insufficient reporting of attrition/exclusions to permit judgment (e.g., number randomized not stated, no reasons for missing data provided) | High  Low  Unclear |  |

* If particular questions/entries were pre-specified in the study's protocol, responses should be provided for each question/entry.

† It is likely that the majority of studies will fall into this category.

Assess each main or class of outcomes for each of the following. Indicate the specific outcome.

## Appendix 2.2: The assessment judgment outcomes of RCTs

| Author name | Year | Title | Sequence generation | Description | Allocation concealment | Description | Blinding of participants, personnel | Description | Blinding of outcome assessors | Description | Incomplete outcome data | Description | Selective outcome reporting | Description | Other sources of bias | Description | Overall |
| --- | --- | --- | --- | --- | --- | --- | --- | --- | --- | --- | --- | --- | --- | --- | --- | --- | --- |
| Al-Nawaiseh | 2016 | Enhancing Short-Term Recovery After High-Intensity Anaerobic Exercise | Low Risk | random & crossover, counterbalanced | Unclear Risk | didn’t mention weather about blinding | Unclear Risk | didn’t mention about blinding | Unclear Risk | didn’t mention weather about blinding | Low Risk | No missing outcome data or loss to follow-up | Low risk | The study protocol is available and all of the study’s pre-specified outcomes of interest have been reported in the pre-specified way | Low risk | The study appears to be free of other sources of bias. | Unclear Risk of Bias |
| Breen | 2011 | The influence of carbohydrate-protein co-ingestion following endurance exercise on myofibrillar and mitochondrial protein synthesis | Low Risk | random & counter-balanced | High Risk | Single Blinding (participants or investigators enrolling participants could possibly foresee assignments) | Low Risk | Although it is single blinding whereby participants or investigators enrolling participants could possibly foresee assignments. However, there is no incomplete blinding, but in the reviewer’s judgment the outcome is not likely to be influenced by lack of blinding | Low Risk | Although it is single blinding whereby participants or investigators enrolling participants could possibly foresee assignments. However, there is no incomplete blinding, but in the reviewer’s judgment the outcome is not likely to be influenced by lack of blinding | Low Risk | No missing outcome data or loss to follow-up | Low risk | The study protocol is available and all of the study’s pre-specified outcomes of interest have been reported in the pre-specified way | Low risk | The study appears to be free of other sources of bias. | High Risk of Bias |
| Coombes | 2002 | Dose effects of oral bovine colostrum on physical work capacity in cyclists. / Effets de la prise orale de colostrum bovin sur les capacites physiques de travail chez des cyclistes | Low Risk | random & placebo-controlled study | Low Risk | Double Blinding | Low Risk | Double Blinding | Low Risk | Double Blinding | Low Risk | No missing outcome data or loss to follow-up | Low risk | The study protocol is available and all of the study’s pre-specified outcomes of interest have been reported in the pre-specified way | Low risk | The study appears to be free of other sources of bias. | Low Risk of Bias |
| Gunnarsson | 2013 | Effect of whey protein- and carbohydrate-enriched diet on glycogen resynthesis during the first 48 h after a soccer game | Unclear Risk | random & without providing the details of what was done | Unclear Risk | didn’t mention weather about blinding | Unclear Risk | didn’t mention about blinding | Unclear Risk | didn’t mention weather about blinding | Low Risk | No missing outcome data or loss to follow-up | Low risk | The study protocol is available and all of the study’s pre-specified outcomes of interest have been reported in the pre-specified way | Low risk | The study appears to be free of other sources of bias. | Unclear Risk of Bias |
| Hansen | 2016 | Protein intake during training sessions has no effect on performance and recovery during a strenuous training camp for elite cyclists | Low Risk | random & block | High Risk | Single Blinding (participants or investigators enrolling participants could possibly foresee assignments) | Low Risk | Although it is single blinding whereby participants or investigators enrolling participants could possibly foresee assignments. However, there is no incomplete blinding, but in the reviewer’s judgment the outcome is not likely to be influenced by lack of blinding | Low Risk | Although it is single blinding whereby participants or investigators enrolling participants could possibly foresee assignments. However, there is no incomplete blinding, but in the reviewer’s judgment the outcome is not likely to be influenced by lack of blinding | Low Risk | No missing outcome data or loss to follow-up | Low risk | The study protocol is available and all of the study’s pre-specified outcomes of interest have been reported in the pre-specified way | Low risk | The study appears to be free of other sources of bias. | High Risk of Bias |
| Highton | 2012 | Carbohydrate-protein coingestion improves multiple-sprint running performance | Low Risk | random & crossover | Low Risk | Double Blinding | Low Risk | Double Blinding | Low Risk | Double Blinding | Low Risk | No missing outcome data or loss to follow-up | Low risk | The study protocol is available and all of the study’s pre-specified outcomes of interest have been reported in the pre-specified way | Low risk | The study appears to be free of other sources of bias. | Low Risk of Bias |
| Hoffman | 2009 | Effect of protein-supplement timing on strength, power, and body-composition changes in resistance-trained men | Unclear Risk | random & without providing the details of what was done | Unclear Risk | didn’t mention weather about blinding | Unclear Risk | didn’t mention about blinding | Unclear Risk | didn’t mention weather about blinding | Low Risk | No missing outcome data or loss to follow-up | Low risk | The study protocol is available and all of the study’s pre-specified outcomes of interest have been reported in the pre-specified way | Low risk | The study appears to be free of other sources of bias. | Unclear Risk of Bias |
| Impey | 2015 | Leucine-enriched protein feeding does not impair exercise-induced free fatty acid availability and lipid oxidation: beneficial implications for training in carbohydrate-restricted states | Low Risk | random & counter-balanced (Latin Squares approach) | Unclear Risk | didn’t mention weather about blinding | Unclear Risk | didn’t mention about blinding | Unclear Risk | didn’t mention weather about blinding | Low Risk | No missing outcome data or loss to follow-up | Low risk | The study protocol is available and all of the study’s pre-specified outcomes of interest have been reported in the pre-specified way | Low risk | The study appears to be free of other sources of bias. | Unclear Risk of Bias |
| Joy | 2013 | The effects of 8 weeks of whey or rice protein supplementation on body composition and exercise performance | Unclear Risk | random & without providing the details of what was done | Low Risk | Double Blinding | Low Risk | Double Blinding | Low Risk | Double Blinding | Low Risk | No missing outcome data or loss to follow-up | Low risk | The study protocol is available and all of the study’s pre-specified outcomes of interest have been reported in the pre-specified way | Low risk | The study appears to be free of other sources of bias. | Unclear Risk of Bias |
| Li | 2007 | Effects of carbohydrate and whey protein supplement at appropriate time on physical performance during football game. [Chinese] | Unclear Risk | random & without providing the details of what was done | Unclear Risk | didn’t mention weather about blinding | Unclear Risk | didn’t mention about blinding | Unclear Risk | didn’t mention weather about blinding | Low Risk | No missing outcome data or loss to follow-up | Low risk | The study protocol is available and all of the study’s pre-specified outcomes of interest have been reported in the pre-specified way | Low risk | The study appears to be free of other sources of bias. | Unclear Risk of Bias |
| Lollo | 2011 | Physiological and physical effects of different milk protein supplements in elite soccer players | Unclear Risk | random & without providing the details of what was done | Low Risk | Double Blinding | Low Risk | Double Blinding | Low Risk | Double Blinding | Low Risk | No missing outcome data or loss to follow-up | Low risk | The study protocol is available and all of the study’s pre-specified outcomes of interest have been reported in the pre-specified way | Low risk | The study appears to be free of other sources of bias. | Unclear Risk of Bias |
| Lollo | 2014 | Hydrolysed whey protein reduces muscle damage markers in Brazilian elite soccer players compared with whey protein and maltodextrin. A twelve-week in-championship intervention | Unclear Risk | random & without providing the details of what was done | Low Risk | Double Blinding | Low Risk | Double Blinding | Low Risk | Double Blinding | Low Risk | No missing outcome data or loss to follow-up | Low risk | The study protocol is available and all of the study’s pre-specified outcomes of interest have been reported in the pre-specified way | Low risk | The study appears to be free of other sources of bias. | Unclear Risk of Bias |
| Macdermid | 2006 | A whey-supplemented, high-protein diet versus a high-carbohydrate diet: effects of endurance cycling performance | Low Risk | random & balanced order | Unclear Risk | didn’t mention weather about blinding | Unclear Risk | didn’t mention about blinding | Unclear Risk | didn’t mention weather about blinding | Low Risk | No missing outcome data or loss to follow-up | Low risk | The study protocol is available and all of the study’s pre-specified outcomes of interest have been reported in the pre-specified way | Low risk | The study appears to be free of other sources of bias. | Unclear Risk of Bias |
| Naclerio | 2015 | A multi-ingredient containing carbohydrate, proteins L-glutamine and L-carnitine attenuates fatigue perception with no effect on performance, muscle damage or immunity in soccer players | Low Risk | random & counter balanced, cross over | Low Risk | Double Blinding | Low Risk | Double Blinding | Low Risk | Double Blinding | Low Risk | No missing outcome data or loss to follow-up | Low risk | The study protocol is available and all of the study’s pre-specified outcomes of interest have been reported in the pre-specified way | Low risk | The study appears to be free of other sources of bias. | Low Risk of Bias |
| Oosthuyse | 2016 | Comparison of energy supplements during prolonged exercise for maintenance of cardiac function: carbohydrate only versus carbohydrate plus whey or casein hydrolysate | Low Risk | random & four way crossover | Low Risk | Double Blinding | Low Risk | Double Blinding | Low Risk | Double Blinding | Low Risk | No missing outcome data or loss to follow-up | Low risk | The study protocol is available and all of the study’s pre-specified outcomes of interest have been reported in the pre-specified way | Low risk | The study appears to be free of other sources of bias. | Low Risk of Bias |
| Schroer | 2014 | Cycling Time Trial Performance May Be Impaired by Whey Protein and L-Alanine Intake During Prolonged Exercise | Low Risk | random & counterbalanced, placebo-controlled | Low Risk | Double Blinding | Low Risk | Double Blinding | Low Risk | Double Blinding | Low Risk | No missing outcome data or loss to follow-up | Low risk | The study protocol is available and all of the study’s pre-specified outcomes of interest have been reported in the pre-specified way | Low risk | The study appears to be free of other sources of bias. | Low Risk of Bias |
| Shing | 2006 | The influence of bovine colostrum supplementation on exercise performance in highly trained cyclists | Low Risk | random & placebo controlled | Low Risk | Double Blinding | Low Risk | Double Blinding | Low Risk | Double Blinding | Low Risk | No missing outcome data or loss to follow-up | Low risk | The study protocol is available and all of the study’s pre-specified outcomes of interest have been reported in the pre-specified way | Low risk | The study appears to be free of other sources of bias. | Low Risk of Bias |
| Smith | 2010 | The effects of a pre-workout supplement containing caffeine, creatine, and amino acids during three weeks of high-intensity exercise on aerobic and anaerobic performance | Low Risk | random & placebo controlled parallel | High Risk | Single Blinding (participants or investigators enrolling participants could possibly foresee assignments) | Low Risk | Although it is single blinding whereby participants or investigators enrolling participants could possibly foresee assignments. However, there is no incomplete blinding, but in the reviewer’s judgment the outcome is not likely to be influenced by lack of blinding | Low Risk | Although it is single blinding whereby participants or investigators enrolling participants could possibly foresee assignments. However, there is no incomplete blinding, but in the reviewer’s judgment the outcome is not likely to be influenced by lack of blinding | Low Risk | No missing outcome data or loss to follow-up | Low risk | The study protocol is available and all of the study’s pre-specified outcomes of interest have been reported in the pre-specified way | Low risk | The study appears to be free of other sources of bias. | High Risk of Bias |
| Taylor | 2016 | Eight weeks of pre- and postexercise whey protein supplementation increases lean body mass and improves performance in Division III collegiate female basketball players | Unclear Risk | random & without providing the details of what was done | Low Risk | Double Blinding | Low Risk | Double Blinding | Low Risk | Double Blinding | Low Risk | No missing outcome data or loss to follow-up | Low risk | The study protocol is available and all of the study’s pre-specified outcomes of interest have been reported in the pre-specified way | Low risk | The study appears to be free of other sources of bias. | Unclear Risk of Bias |
| Vegge | 2012 | Improved cycling performance with ingestion of hydrolyzed marine protein depends on performance level | Low Risk | random & crossedover | Low Risk | Double Blinding | Low Risk | Double Blinding | Low Risk | Double Blinding | Low Risk | No missing outcome data or loss to follow-up | Low risk | The study protocol is available and all of the study’s pre-specified outcomes of interest have been reported in the pre-specified way | Low risk | The study appears to be free of other sources of bias. | Low Risk of Bias |

# Appendix 3: Results of pairwise meta-analysis

For abbreviations all tables, WPS represent whey protein supplements; WMD represent weighted mean differences.

When WMD higher than 0, it is favour to the intervention which is whey protein supplements.

| **Comparisons** | | **Pairwise meta-analysis WMD (95% CI)** | **No. of participants** | **No. of trials** | **Heterogeneity I-squared (**$\boldsymbol{I}^{\boldsymbol{2}}$**) (the variation in WMD attributable to heterogeneity)** |
| --- | --- | --- | --- | --- | --- |
| Comparators | Intervention |  |  |  |  |
| **Heart rate (bpm)** | | | | | |
| Carbohydrate vs. | WPS | -0.803 (-2.130 to 0.524) | 70 | 4 | 0.0% |
| Placebo vs. |  | **2.004 (1.297 to 2.711)** | 48 | 3 | 0.0% |
| Protein+caffeine vs. |  | -1.000 (-2.089 to 0.089) | 18 | 1 | Not Applicable (NA) |
| Casein vs. |  | 2.000 (-5.366 to 9.366) | 16 | 1 | NA |
| L-alanine vs. |  | 5.000 (-5.231 to 15.231) | 16 | 1 | NA |
| Maltodextrin vs. |  | 1.000 (-5.359 to 7.359) | 48 | 2 | 0.0% |
| **Respiratory exchange ratio (RER)** | | | | | |
| Carbohydrate vs. | WPS | 0.000 (-0.004 to 0.004) | 20 | 1 | NA |
| L-alanine vs. |  | 0.000 (-0.029 to 0.029) | 16 | 1 | NA |
| Placebo vs. |  | 0.010 (-0.015 to 0.035) | 16 | 1 | NA |
| Maltodextrin vs. |  | **0.012 (0.001 to 0.023)** | 48 | 2 | 0.0% |
| **Rate perceived exertion (RPE)** | | | | | |
| Carbohydrate vs. | WPS | -0.044 (-1.376 to 1.287) | 70 | 3 | 88.9% |
| Leucine vs. |  | 1.000 (0.692 to 1.308) | 18 | 1 | NA |
| Placebo vs. |  | **-****1.056 (-1.816 to -0.296)** | 66 | 3 | 62.5% |
| L-alanine vs. |  | 0.000 (-1.274 to 1.274) | 16 | 1 | NA |
| **Maximum volume of oxygen (**$\boldsymbol{VO}_{\mathbf{2}\boldsymbol{max}}$**) (ml/kg/min)** | | | | | |
| Carbohydrate vs. | WPS | 0.000 (-0.643 to 0.643) | 20 | 1 | NA |
| Bovine colostrum vs. |  | -2.658 (-6.180 to 0.865) | 66 | 3 | 0.0% |
| L-alanine vs. |  | 60.000 (-238.935 to 358.935) | 16 | 1 | NA |
| Placebo vs. |  | 30.000 (-264.158 to 324.158) | 16 | 1 | NA |
| Maltodextrin vs. |  | 4.064 (-4.230 to 12.359) | 72 | 3 | 97.8% |
| **Maximum Power (watt)** | | | | | |
| Placebo vs. | WPS | 66.281 (-4.275 to 136.837) | 84 | 3 | 0.0% |
| Carbohydrate vs. |  | -88.935 (-303.233 to 125.364) | 48 | 3 | 95.9% |
| Rice vs. |  | 31.100 (-55.790 to 117.990) | 24 | 1 | NA |
| Bovine colostrum vs. |  | 14.000 (-13.303 to 41.303) | 29 | 1 | NA |
| **Average Power (watt)** | | | | | |
| Placebo vs. | WPS | 10.559 (-32.207 to 53.326) | 84 | 3 | 0.0% |
| Bovine colostrum vs. |  | -64.525 (-155.644 to 26.593) | 37 | 2 | 67.7% |
| Carbohydrate vs. |  | -2.057 (-6.930 to 2.816) | 66 | 4 | 76.6% |
| **Body Mass (kg)** | | | | | |
| Bovine colostrum vs. | WPS | 0.585 (-6.122 to 7.292) | 37 | 2 | 0.0% |
| Placebo vs. |  | -1.071 (-16.884 to 14.743) | 40 | 2 | 0.0% |
| Casein vs. |  | -5.593 (-8.131 to -3.054) | 32 | 2 | 86.0% |
| Maltodextrin vs. |  | -2.971 (-5.045 to -0.896) | 48 | 3 | 0.0% |
| Carbohydrate vs. |  | 0.200 (-26.064 to 26.464) | 14 | 1 | NA |

# Appendix 4: Network meta-analysis plots

The size of the nodes corresponds or represents the number of trials. The directly compared supplements are linked with a line, the thickness of which corresponds or represents the number of trials that assessed this between supplements.

1. **Heart rate**


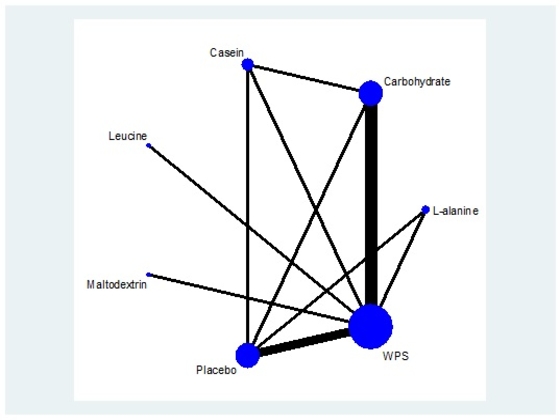


1. **RER**


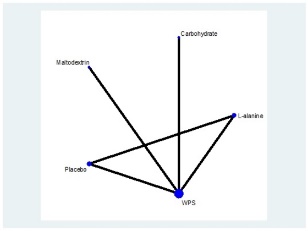


1. **RPE**

**
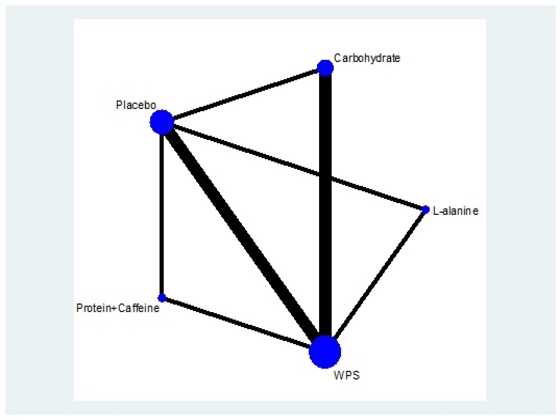
**

1. $\boldsymbol{VO}_{\mathbf{2}\boldsymbol{max}}$


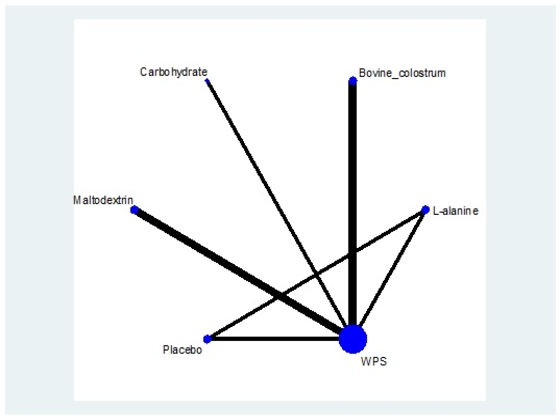


1. **Maximum Power**

**
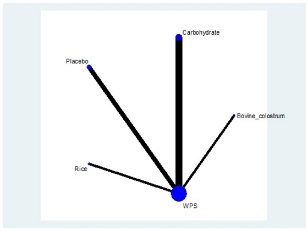
**

1. **Average Power**

**
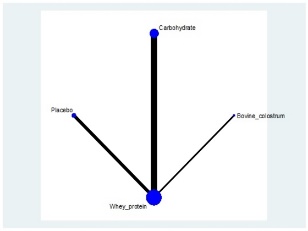
**

1. **Body Mass**

**
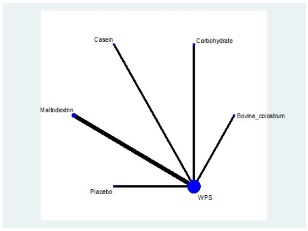
**

# Appendix 5: Assessment of inconsistency for each outcome network meta-analysis

Assessment of inconsistency in treatment triangular or quadratic loops for each outcome network as the difference between direct and indirect estimates (called inconsistency factor (IF)). Estimated inconsistency with the corresponding of 95% for each IF in each closed triangular or quadratic loop. The following table show closed triangular loops (formed by three supplement) in each outcome network. Inconsistent loops are presented IF of 95% confidence intervals incompatible with zero. For example, the triangular loop of evidence including supplementation comparison between carbohydrate, combined casein and WPS (Carbohydrate – casein – WPS), the WMD for estimated effect differs between direct and indirect evidence by 3.913, yet the 95% confidence interval includes 0 indicating the possibility of no difference.

| **Closed triangular or quadratic loops of evidence** | **Inconsistency factor (95% CI)** | **Loop heterogeneity,** $\boldsymbol{\tau}^{\boldsymbol{2}}$ |
| --- | --- | --- |
| **Heart rate** | | |
| Carbohydrate – casein – WPS | 3.913 (0.00,15.12) | 0.000 |
| Carbohydrate – placebo – WPS | 2.917 (0.00,15.13) | 0.000 |
| Casein – placebo – WPS | 1.000 (0.00,14.91) | 0.000 |
| L-alanine – placebo – WPS | 0.004 (0.00,14.49) | 0.000 |
| Carbohydrate – casein – placebo | 0* | 0.000 |
| **RER** | | |
| L-alanine – placebo – WPS | 0* | 0.000 |
| **RPE** | | |
| L-alanine – placebo – WPS | 0.982 (0.00,2.77) | 0.000 |
| Carbohydrate -placebo - WPS | 0.863 (0.00,3.22) | 0.412 |
| Placebo – protein+caffeine – WPS | 0.207 (0.00,1.08) | 0.000 |
| $\boldsymbol{VO}_{\mathbf{2}\boldsymbol{max}}$ **(ml/kg/min)** | | |
| L-alanine – placebo – WPS | 0* | 0.000 |
| **Maximum Power** | | |
| No triangular or quadratic loops found | | |
| **Average Power** | | |
| No triangular or quadratic loops found | | |
| **Body Mass** | | |
| No triangular or quadratic loops found | | |

*Heterogeneity of loop could not be estimated due to insufficient observations and was set equal to 0.

# Appendix 6: Assessment of global inconsistency in networks using the ‘design-by-treatment’ interaction model

| **Network outcome** | **Chi-square (**$\boldsymbol{\chi}^{\boldsymbol{2}}$**)** | **P value for test of global inconsistency** |
| --- | --- | --- |
| Heart rate | 0.92 | 0.8202 |
| RER | 2.40 | 0.1213 |
| RPE | 3.17 | 0.3667 |
| ${VO}_{2max}$ | 1.31 | 0.2526 |
| Maximum Power | 0.05 | 0.8253 |
| Average Power | 0.00 | 0.9699 |
| Body Mass | 0.02 | 0.8931 |

# Appendix 7: SUCRA ranks

1. **Heart rate**

| **Supplements** | **SUCRA (%)** | **SUCRA rank** | **Estimated probabilities (PrBest) (%)** | **Estimated probabilities (PrBest) rank** | **Estimate Cumulative Probability (MeanRank) (%)** | **Estimate Cumulative Probability (MeanRank) rank** |
| --- | --- | --- | --- | --- | --- | --- |
| Carbohydrate | 74.9 | 1 | 38.4 | 1 | 2.5 | 7 |
| Casein | 53.3 | 3 | 19.9 | 2 | 3.8 | 5 |
| L-alanine | 59.6 | 2 | 8.7 | 5 | 3.4 | 6 |
| Leucine | 46.1 | 5 | 11.3 | 4 | 4.2 | 3 |
| Maltodextrin | 33.9 | 6 | 2.3 | 6 | 5.0 | 1 |
| Placebo | 49.1 | 4 | 19.5 | 3 | 4.1 | 4 |
| WPS | 33.1 | 7 | 0.0 | 7 | 5.0 | 1 |

1. **RER**

| **Supplements** | **SUCRA (%)** | **SUCRA rank** | **Estimated probabilities (PrBest) (%)** | **Estimated probabilities (PrBest) rank** | **Estimate Cumulative Probability (MeanRank) (%)** | **Estimate Cumulative Probability (MeanRank) rank** |
| --- | --- | --- | --- | --- | --- | --- |
| Carbohydrate | 48.8 | 4 | 7.8 | 4 | 3.0 | 2 |
| L-alanine | 71.1 | 1 | 13.6 | 3 | 2.4 | 5 |
| Maltodextrin | 57.5 | 3 | 35.7 | 2 | 2.7 | 3 |
| Placebo | 42.8 | 5 | 2.5 | 5 | 3.3 | 1 |
| WPS | 59.1 | 2 | 40.4 | 1 | 2.6 | 4 |

1. **RPE**

| **Supplements** | **SUCRA (%)** | **SUCRA rank** | **Estimated probabilities (PrBest) (%)** | **Estimated probabilities (PrBest) rank** | **Estimate Cumulative Probability (MeanRank) (%)** | **Estimate Cumulative Probability (MeanRank) rank** |
| --- | --- | --- | --- | --- | --- | --- |
| Carbohydrate | 36.4 | 3 | 3.7 | 3 | 3.5 | 3 |
| L-alanine | 31.0 | 5 | 0.1 | 5 | 3.8 | 1 |
| Placebo | 57.0 | 2 | 12.9 | 2 | 2.7 | 4 |
| Protein+Caffeine | 93.0 | 1 | 82.2 | 1 | 1.3 | 5 |
| WPS | 32.7 | 4 | 1.1 | 4 | 3.7 | 2 |

1. $\boldsymbol{VO}_{\mathbf{2}\boldsymbol{max}}$

| **Supplements** | **SUCRA (%)** | **SUCRA rank** | **Estimated probabilities (PrBest) (%)** | **Estimated probabilities (PrBest) rank** | **Estimate Cumulative Probability (MeanRank) (%)** | **Estimate Cumulative Probability (MeanRank) rank** |
| --- | --- | --- | --- | --- | --- | --- |
| L-alanine | 49.6 | 3 | 14.2 | 3 | 3.5 | 4 |
| Bovine colostrum | 63.5 | 2 | 60.3 | 1 | 2.8 | 5 |
| Carbohydrate | 29.8 | 6 | 2.6 | 5 | 4.5 | 1 |
| Maltodextrin | 44.7 | 4 | 5.3 | 4 | 3.8 | 2 |
| Placebo | 69.4 | 1 | 16.6 | 2 | 2.5 | 6 |
| WPS | 43.0 | 5 | 0.9 | 6 | 3.8 | 3 |

1. **Maximum Power**

| **Supplements** | **SUCRA (%)** | **SUCRA rank** | **Estimated probabilities (PrBest) (%)** | **Estimated probabilities (PrBest) rank** | **Estimate Cumulative Probability (MeanRank) (%)** | **Estimate Cumulative Probability (MeanRank) rank** |
| --- | --- | --- | --- | --- | --- | --- |
| Bovine colostrum | 70.7 | 1 | 38.3 | 1 | 2.2 | 5 |
| Carbohydrate | 53.5 | 3 | 25.3 | 3 | 2.9 | 3 |
| Placebo | 17.9 | 5 | 1.8 | 5 | 4.3 | 1 |
| Rice | 57.9 | 2 | 30.4 | 2 | 2.7 | 4 |
| WPS | 50.0 | 4 | 4.2 | 4 | 3.0 | 2 |

1. **Average Power**

| **Supplements** | **SUCRA (%)** | **SUCRA rank** | **Estimated probabilities (PrBest) (%)** | **Estimated probabilities (PrBest) rank** | **Estimate Cumulative Probability (MeanRank) (%)** | **Estimate Cumulative Probability (MeanRank) rank** |
| --- | --- | --- | --- | --- | --- | --- |
| Bovine colostrum | 70.6 | 2 | 51.3 | 1 | 1.9 | 3 |
| Carbohydrate | 0.2 | 4 | 0.1 | 4 | 4.0 | 1 |
| Placebo | 53.8 | 3 | 10.2 | 3 | 2.4 | 2 |
| WPS | 75.4 | 1 | 38.4 | 2 | 1.7 | 4 |

1. **Body Mass**

| **Supplements** | **SUCRA (%)** | **SUCRA rank** | **Estimated probabilities (PrBest) (%)** | **Estimated probabilities (PrBest) rank** | **Estimate Cumulative Probability (MeanRank) (%)** | **Estimate Cumulative Probability (MeanRank) rank** |
| --- | --- | --- | --- | --- | --- | --- |
| Bovine colostrum | 59.0 | 3 | 32.8 | 1 | 3.0 | 4 |
| Carbohydrate | 66.9 | 1 | 27.5 | 3 | 2.7 | 6 |
| Casein | 53.2 | 4 | 31.4 | 2 | 3.3 | 3 |
| Maltodextrin | 12.4 | 6 | 0.0 | 6 | 5.4 | 1 |
| Placebo | 43.8 | 5 | 0.9 | 5 | 3.8 | 2 |
| WPS | 64.7 | 2 | 7.4 | 4 | 2.8 | 5 |
